# Mouse Models of Down Syndrome as a Tool to Unravel the Causes of Mental Disabilities

**DOI:** 10.1155/2012/584071

**Published:** 2012-05-22

**Authors:** Noemí Rueda, Jesús Flórez, Carmen Martínez-Cué

**Affiliations:** Department of Physiology and Pharmacology, Faculty of Medicine, University of Cantabria, Santander 39011, Spain

## Abstract

Down syndrome (DS) is the most common genetic cause of mental disability. Based on the homology of Hsa21 and the murine chromosomes Mmu16, Mmu17 and Mmu10, several mouse models of DS have been developed. The most commonly used model, the Ts65Dn mouse, has been widely used to investigate the neural mechanisms underlying the mental disabilities seen in DS individuals. A wide array of neuromorphological alterations appears to compromise cognitive performance in trisomic mice. Enhanced inhibition due to alterations in GABA_A_-mediated transmission and disturbances in the glutamatergic, noradrenergic and cholinergic systems, among others, has also been demonstrated. DS cognitive dysfunction caused by neurodevelopmental alterations is worsened in later life stages by neurodegenerative processes. A number of pharmacological therapies have been shown to partially restore morphological anomalies concomitantly with cognition in these mice. In conclusion, the use of mouse models is enormously effective in the study of the neurobiological substrates of mental disabilities in DS and in the testing of therapies that rescue these alterations. These studies provide the basis for developing clinical trials in DS individuals and sustain the hope that some of these drugs will be useful in rescuing mental disabilities in DS individuals.

## 1. Introduction

Trisomy 21, or Down syndrome (DS), is the most common genetic cause of intellectual disability. It affects 1 in 850–1000 infants [1] and is characterized by a number of phenotypes, including cardiovascular, skeletal, and motor alterations. However, the most prominent feature of DS is an intellectual disability that affects 100% of the individuals with this condition. DS individuals typically display an average Intelligence Quotient (IQ) of 50 (ranging from 30 to 70) [2] and show an array of altered cognitive and behavioral phenotypes, including the incomplete and delayed acquisition of motor [3], linguistic [3, 4] and visual-spatial abilities [3], impairments in learning and memory [3–6], and neurobehavioral disorders [4] and have a higher risk of developing Alzheimer-like dementia by the age of 40 [7, 8]. Great interindividual variability, however, is present in both the nature and the intensity of all of these conditions.

In recent years, the question of how trisomy of Hsa21 leads to this set of phenotypes has been a matter of debate. Two hypotheses have been proposed to account for this phenomenon: the “amplified developmental instability” hypothesis [9] and the “gene-dosage effect” hypothesis [10–12]. The first hypothesis proposes that trisomy of Hsa21 causes a general alteration in developmental homeostasis that leads to the DS phenotypes; the “gene-dosage effect” proposal maintains that these alterations result from the overexpression of a subset of genes and their encoded proteins.

The analysis of DS cases resulting from partial trisomies of Hsa21 and the development of a number of mouse models of this condition have provided insight on the causative role of dosage-sensitive genes on DS phenotypes. These studies have yielded evidence that support both theories; while the role of single dosage-sensitive genes on different phenotypes has been confirmed, research has also demonstrated that many of these DS features are due to the complex effects of multiple Hsa21 genes (see [13]) and their interactions with genes of other chromosomes.

To identify the biological mechanisms underlying different pathologies and to evaluate the efficacy of novel therapies, thousands of animal models of human disorders have been developed. For an animal model of a human disorder to be considered valid, it has to satisfy three criteria: *construct*, *face*, and *predictive* validity [14, 15]. *Construct* validity relates to the similarity between the etiology of the human and the animal disorder (e.g., in the case of mouse models of DS, the triplication of Hsa21 genes). *Face *validity refers to how well the model mimics the molecular, cellular, physiological, and behavioral phenotypes observed in humans. *Predictive* validity requires that the new knowledge obtained in the animal model makes accurate predictions of what will be found in the human condition. This validity is particularly important for unraveling the neurobiological causes of the cognitive deficits found in DS that cannot be assessed in humans for ethical or practical reasons and for developing and testing new therapies.

In the following sections, we will summarize (1) the similarities between the genetic overdose of various mouse models of DS and human trisomy 21; (2) the concordance between the behavioral, neuromorphological, and neurochemical phenotypes of DS mouse models and the human condition; (3) the knowledge obtained in these animals about the neurobiology of DS that have yielded the development and analysis of several therapeutic strategies that could potentially be used to attenuate cognitive impairments in DS individuals.

## 2. Mouse Models of Down Syndrome

**Figure 1:**
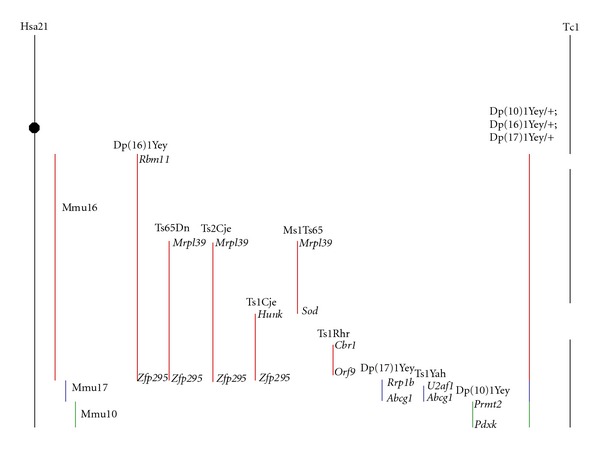
Schematic representation of Hsa21 and syntenic regions of Mmu16, Mmu17, and Mmu10 and the different mouse models trisomic for different sets of genes orthologous to those of Hsa21. The flanking genes found at the boundaries of the triplicated region in each model are written in italics. Modified from [3, 137].

The long arm of Hsa21 contains approximately 552 genes, 166 of which are orthologous to genes localized in syntenic regions of three mouse chromosomes: Mmu16 (110 orthologous genes), Mmu17 (19 orthologous genes), and Mmu10 (37 orthologous genes) [16]. Based on these homologies, several mouse models that are trisomic for different sets of Hsa21 genes have been developed (Figure 1). The first attempt to create a mouse model of DS was to develop a mouse, named Ts16, which was trisomic for the entire Mmu16 [17]. However, this model does not resemble the DS aneuploidy because Mmu16 presents syntenies with regions of Hsa3, Hsa8, Hsa16, and Hsa21; thus, it has triplicated many genes that are not in trisomy in DS and, consequently, does not exhibit good construct validity. Furthermore, Ts16 embryos die in utero, making it impossible to test phenotypes in young and adult mice, thus restricting the face and predictive validities of this model.

The next approach adopted was the generation of mouse models with partial trisomies of sets of Mmu16 genes orthologous to those found in Hsa21. In 1993, Davisson et al. [18] created the Ts65Dn mouse, which is now the most commonly used and best characterized model of DS. This mouse bears a partial trisomy of a segment of Mmu16, extending from the Mrp139 to the Znf295 genes, and contains approximately 92 genes orthologous to Hsa21 genes [16]. Additionally, Ts65Dn mice also carry a trisomy of ~10 Mb of Mmu17 containing 60 genes nonhomologous to Hsa21 [19]. Thus, this model does not have perfect construct validity because many of the orthologous genes found in Hsa21 are not triplicated in this mouse and because a set of genes not triplicated in DS are in trisomy in this model. However, as detailed below, the Ts65Dn mouse is currently the model that displays the best face validity. Additionally, in some cases, DS results from a partial trisomy of different regions of Hsa21, and there is strong evidence that some regions of this chromosome contribute more to the DS phenotype [12, 20]. Moreover, according to the “gene-dosage effect” hypothesis, different DS phenotypes are determined by the increased dosage of only a subset of genes. A comparison of the phenotypes in Ts65Dn mice with those of other partial trisomic models (see below) suggests that the set of genes triplicated in this model contribute to several DS phenotypes, including cognitive and neuroanatomical impairments (Tables 1 and 2).

The Ts2Cje model carries the same segment of Mmu16 triplicated in the Ts65Dn mouse but is translocated to chromosome 12 [21]. Although this model also shows some of the DS-relevant phenotypes found in the Ts65Dn mouse, it has not been fully characterized.

Several other segmental trisomic models of different segments of Mmu16, 17, and 10 have been created. In the late 90s, Sago and coworkers generated two mouse models with the triplication of two different regions of Mmu16: the Ts1Cje mouse, which presents a trisomy of 81 genes localized in the region of Mmu16 that extend from Sod1 to Znf295 [22]; the Ms1Ts65 mouse, which has a partial trisomy of 33 genes mapped in the region of Hsa21 that extend from App to Sod1 [23]. In addition, to evaluate the influence of the so-called Down syndrome critical region (DSCR), Olson et al. [24] developed the Ts1Rhr mouse, a model that is trisomic for the Cbr1-Orf9 region of Mmu16, which contains 33 genes. Finally, Li et al. [25] generated a mouse trisomic for the complete Hsa21 syntenic region on Mmu16 (between Lipi and Zfp295) containing 110 orthologous genes, the Dp(16)1Yey/+ mouse [16].

To model the trisomy of Hsa21 orthologous genes located in Mmu17, two mouse models have been created: the Ts1Yah mouse, trisomic for 12 genes in the Mmu17 region, syntenic to the subtelomeric region of Hsa21 [26] and the Dep(17)1Yey/+ mouse which is trisomic for the entire Hsa21 syntenic region on Mmu17 that contains 19 orthologous genes [16, 27, 28]. Additionally, Vacík et al. [29] created the Ts43H model, a mouse that is trisomic for 30 Mb of Mmu17 containing over 300 genes but only ~20 of them are orthologs of Hsa21 genes. Therefore, this is not a valid DS mouse model.

The last segmental trisomic mouse generated is a mouse that models the trisomy of Hsa21 orthologous genes located in Mmu10. The Dp(10)1Yey mouse is trisomic for the region of Mmu10 syntenic to the distal part of Hsa21 containing 37 orthologous genes [16, 27].

After the tree partial trisomic models for all the Hsa21 syntenic regions on Mmu10 (Dp(10)1Yey/+), Mmu16 (Dp(16)1Yey/+) and Mmu17 (Dep(17)1Yey/+) were established, Yu et al. [27] cross-breeded them to generate a mouse that is trisomic for the entire Hsa21 syntenic regions on Mmu10, Mmu16, and Mmu17 chromosomes: the Dp(10)1Yey/+Dp(16)1Yey/+; Dep(17)1Yey/+ mouse. This is a promising new model with excellent construct and face validities, as it shows several DS phenotypes [27].

The Tc1 model is a mouse in which the entire human Hsa21 has been triplicated [30]. This mouse shows different DS-relevant phenotypes [30–32], although its characterization is not as complete as those performed on the different segmental trisomic models. However, the Tc1 mouse presents variable levels of mosaicism of the extra chromosome in different tissues, confounding the analysis of phenotypic consequences. In addition, although the starting material was an intact Hsa21, delections occurred and this mouse has triplicated ~83% of the genes on Hsa21.

Finally, to study the role of particular genes in the DS phenotypes, a number of mouse models with the triplication of single genes and trisomic mice in which the expression of only one of the genes triplicated in DS have been normalized have been created (see [13]).

## 3. Cognitive and Behavioral Deficits in Mouse Models of DS

This section describes the similarities between the cognitive and behavioral disturbances found in various mouse models of DS compared to the human condition (Table 1).

Motor dysfunction is a hallmark of DS. Hypotonia, hyporeflexia, reduced muscular strength, disturbances in striate muscle control, and delays in the acquisition of fine and gross motor skills are found in DS individuals from early childhood [33–35].

Ts65Dn mice are not impaired in sensorimotor abilities such as forelimb strength, postural skills, equilibrium, and climbing [36, 37]. However, these mice show poorer balance and motor coordination [38]. Ts65Dn mice are hyperactive in the dark [36, 39, 40] and in other settings that provoke caution and lack of movement in normal animals, such as in open-field and plus-maze tests [36, 41–43]. This hyperactivity has been proposed to be due to a failure to inhibit activity or as a deficit in the ability to attend to relevant stimuli [44–46]. Attention deficits in Ts65Dn mice have been confirmed by Driscoll et al. [47]. Unlike Ts65Dn, the other models that are partially trisomic for different segments of Mmu16 are not hyperactive. Ts1Cje and Ms1Ts65 mice do not exhibit altered spontaneous activity [23], and Ts1Rhr mice display normal performances in the open-field test [48]. However, Tc1 mice present higher spontaneous locomotor activity, reduced ability to habituate to new environments, and several deficits of motor coordination and balance in the rotarod and static-rod tests [32].

Similar to DS [6], Ts65Dn mice are impaired in performing hippocampal-dependent tasks, such as spontaneous alterations in the T-maze, contextual fear conditioning, novel object recognition [49, 50], and spatial memory in the radial arm maze [51–54] and the Morris water maze tests [23, 40, 42, 45, 55]. Ts65Dn mice also show deficits in learning an operant conditioning paradigm [56].

Ts1Cje and Ms1Ts65 mice display poor performance in hippocampal-dependent tasks such as the T-maze [52] and the Morris water maze [22, 23]. Ts1Rhr mice are impaired in spontaneous alterations in the T-maze task [48] and show altered long-term memory in the novel object recognition test [48] but not in the Morris water maze [57]. Dp(16)1Yey/+ mice showed impaired performance in the Morris water maze and in the contextual fear conditioning test [27].

Regarding the two mouse models trisomic for segments of Mmu17, Ts1Yah mice are impaired in the novel object recognition and Y-maze test, but their performance in the Morris water maze is enhanced [26]; however, Dp(17)1Yey+ do not show alterations in performance in the later task or in the contextual fear conditioning test [28].

The Dp(10)1Yey/+Dp(16)1Yey/+; Dep(17)1Yey/+ mouse is impaired in the Morris water maze and in the contextual fear conditioning test [27]. Finally, Tc1 mice show altered performance in the novel object recognition test but not in the T-maze [30].

In summary, most of the above-mentioned mouse models show characteristic DS cognitive and behavioral phenotypes, although they differ in the degree of impairment.

## 4. Neuromorphological Alterations in DS and DS Mouse Models

Several mechanisms have been proposed to be the neurobiological correlates of intellectual disability in DS, including altered neurogenesis, hypocellularity, altered synaptic development, increased inhibition, and neurodegeneration.  Table 2 summarizes the main neuromorphological and electrophysiological alterations found in DS individuals and in the different mouse models of DS, and Table 3 describes the outcome of several studies that tested the ability of different therapeutical approaches to rescue different phenotypic alterations in the Ts65Dn mouse and in DS individuals.

### 4.1. Reduced Volume and Hypocellularity

In DS individuals, the volume of the brain is reduced, beginning at early developmental stages [57–62]; in adults, the reduction in size reaches approximately 20% [63, 64], and, during aging, neurodegeneration further deteriorates this scenario [65].

The brain volume of Ts65Dn, Ts1Cje, and Ts2Cje mice is also reduced during the embryonic period, but not after birth or during adulthood [39, 52, 66–68]. Ms1Rhr mice also show reduced brain volume [66], as does the Ts1Rhr mouse, at 4 months of age [69] but not at later stages [48]. Thus, most DS mouse models do not show changes in total brain volume during adulthood.

A number of studies demonstrated that brain areas are dissimilarly affected. Reduced volumes of the hippocampus, entorhinal, frontal, prefrontal, and temporal cortices, amygdala, cerebellum, brain stem nuclei, and mammillary bodies of the hypothalamus have been reported in children and adults with DS [63, 64, 70–78].

Consistently, size and anatomical alterations have been found in some brain regions of trisomic mice. The hippocampus and the cerebellum appear to be the most affected structures. Spatial learning is known to depend on the functional integrity of the hippocampus, a structure that plays a key role in information encoding and retrieving in the CNS [79, 80]. In Ts65Dn mice, the hippocampal granule cell layer and hilus show reduced volume [54, 81–83]. The hippocampal area of Ts1Cje mouse is not reduced [52], and the Ts1Rhr mice show greater volume of the posterior hippocampus [48].

In DS individuals, cell density is not compromised in early gestation [60, 62, 84], but neuron number is reduced in late gestation (after weeks 19–23). Indeed, the number of neurons in the hippocampus, parahippocampal gyrus, cerebellum and neocortex of fetuses [60, 61, 85], and newborn DS individuals [60, 62, 84] is reduced. Hypocellularity persists in different areas of the brain [86] and cerebellum [37] of children and adults with DS.

Ts65Dn mice show reduced cell density during prenatal (E 18.5) and early postnatal stages in the neocortex (P8) [67]. At 1 month of age, Ts65Dn mice display a normal number of neurons in the hippocampal CA1–CA3 areas [81]. However, CA1 neuron density is lower in older (17-18 month) Ts65Dn mice [87, 88]. The dentate gyrus (DG) of Ts65Dn mice has fewer granule cells at all examined ages [54, 81, 87, 89, 90]. However, in 18-month-old Ts1Cje mice, the thickness of the granule cell layer and molecular layer of the DG is not affected [52].

The cerebellum has been traditionally implicated in motor coordination, but there is increasing evidence for the role of this structure in higher cognitive processes, including attention, cognitive flexibility, and memory [91]. Consistent with what is found in DS, the volume of the cerebellum is significantly reduced in Ts65Dn, Ts1Cje, and Ts1Rhr mice [37, 48, 66, 92]. However, Ms1Ts65 mice do not show changes in cerebellar volume [92].

As expected from the reduced cerebellar volume of DS and trisomic mice, the cerebellum of Ts65Dn mice has a lower number of granule and Purkinje cells from early postnatal stages to adulthood [37, 93, 94]. Ts1Cje, Ms1Rs65, and Tc1 mice also show a decrease in cerebellar granule cell density [30, 92].

One of the anatomical substrates of learning and attention is the septohippocampal cholinergic system [95, 96]. In the aging DS brain, there is a loss of cholinergic neurons [97, 98]. Several studies have demonstrated an age-dependent decline in cholinergic markers in Ts65Dn mice. Starting at 6 months of age, Ts65Dn mice basal forebrain cholinergic neurons show a loss of the cholinergic phenotype. These neurons show a decrease in choline acetyltransferase (ChAT) and p75^NGFR^, a neurotrophin receptor localized in basal forebrain cholinergic neurons, immunoreactivity. Furthermore, there is a decrease in the size of these cholinergic neurons cell bodies [82, 99–102].

In conclusion, persistent hypocellularity is one cause of intellectual disability in the trisomic condition. The following sections will describe the evidence showing that these neuroanatomical alterations could be caused by impaired cell proliferation, increased apoptosis and/or neurodegeneration.

### 4.2. Neurogenesis

#### 4.2.1. Neurogenesis in Trisomic States

Neurogenesis is severely compromised in DS from early developmental stages. Impaired neuronal precursor proliferation, slowing of the cell cycle, and altered differentiation are thought to account for altered neurogenesis.

In DS fetuses, a reduced number of dividing cells is found in the dentate gyrus (DG) and lateral ventricle [60, 89]. Reduced proliferation of neural precursor cells is also found in mouse models of DS. In Ts65Dn mice, reduced neural precursor proliferation is found in the neocortical ventricular zone (VZ) during embryonic stages [67]. However, in these mice, a larger progenitor population of inhibitory neurons has been found in the embryonic medial ganglionic eminence [103].

Reductions in neural progenitor cells and neuroblasts, leading to altered neurogenesis, in the embryonic neocortex and subventricular zone (SVZ) of Ts1Cje and Ts2Cje mice have also been reported [68, 104, 105].

It has been proposed that the extra copy of Hsa21 in DS delays the mitotic cell cycle of neuronal precursors, thereby affecting cell proliferation. Accordingly, a slowing of the cell cycle in different trisomic conditions has been demonstrated. In Ts65Dn mice, the cell cycle is extended during embryonic stages in CA3 [67] and in early postnatal life in the DG [89]. The expression of two regulators of the G2/M and G1/S transitions, Ccnb1 and Skp2, is decreased in newbornTs65Dn cerebellar granule cell precursors [82]. Hewitt et al. [104] also observed dysregulated expression of genes involved in cell cycle control in Ts1Cje mice.

Impaired differentiation also appears to contribute to the smaller number of neurons in DS brains [106, 107]. Ts1Cje neural progenitors have a reduced capacity to differentiate into neurons [104, 105].

**Figure 2:**
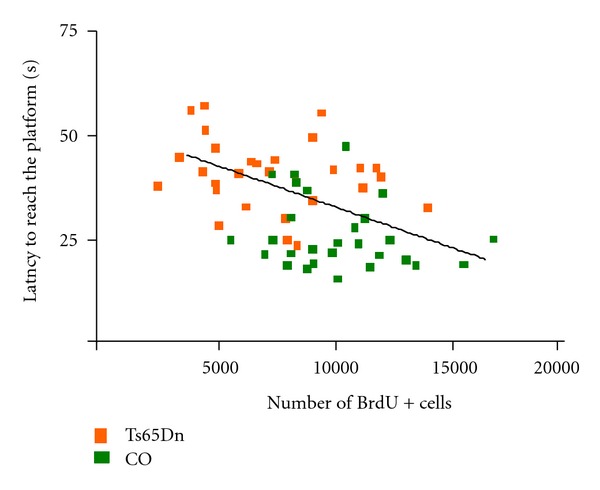
Correlation between performance in the Morris water maze (mean latency to reach the platform) and the number of BrdU+ cells in the DG of Ts65Dn and euploid littermates (Rueda et al., unpublished results; Pearson's *R*: −0.4647; *P* < 0.001).

Adult hippocampal neurogenesis has been demonstrated in many species, including rodents [108–111]. During the entire life span, cell proliferation takes place in the SVZ and in the subgranular zone (SGZ) of the DG, where a pool of multipotent progenitor cells is located. In the SGZ, newborn neurons migrate into the granular cell layer (GCL) and establish functional connections in the dentate molecular region, where they receive excitatory synaptic input from perforant path afferents [111]. Increasing evidence indicates that adult hippocampal neurogenesis is implicated in the establishment of long-term potentiation (LTP) and has a role in hippocampal-dependent learning and memory [112–114]. Interestingly, we have showed a negative correlation between performance in the Morris water maze and the number of proliferating cells in the DG of Ts65Dn mice and euploid littermates (Figure 2).

In Ts65Dn mice, cell proliferation in the SVZ is reduced from birth to adulthood [54, 114, 115]. In the DG, proliferation impairments have also been reported in newborn [81, 89], young [54, 116], middle-aged [117], and aged [83] Ts65Dn mice. Adult (3-month-old) Ts1Cje and Ts2Cje mice also present severe neurogenesis reduction in the SGZ [118].

Cerebellar neurogenesis is also affected by trisomy. DS fetuses show reduced neurogenesis in the external granular layer (EGL) of the cerebellum and in the VZ [61]. Newborn (P0, P2, and P6) Ts65Dn mice also show reduced proliferation of cerebellar granule cell precursors in the EGL [93, 119]; their cell cycle is also dramatically slowed, and the G1 and G2 phases are the most affected [119]. One-month-old Ts65Dn mice show reduced proliferation of the granule neurons of the internal granular layer [94]. In Ts1Cje mice, proliferation of cerebellar granule cells is reduced at birth but normal at postnatal days 3 and 7 [120]. Differentiation is also altered in the cerebellum of Ts65Dn mice because a smaller percentage of cells acquire a neuronal phenotype [119]. Reductions in cerebellar neurogenesis in Ts65Dn mice seem to be due to the decreased response of granule cell precursors to the mitogenic factor Sonic hedgehog (Shh) [93].

It can be concluded that neurogenesis impairment, due to reductions in neural precursors, cell cycle timing and differentiation, is a hallmark of trisomic conditions from prenatal to adult stages. This altered proliferation is likely to be one of the mechanisms responsible for the widespread hypocellularity leading to altered synaptogenesis, connectivity, synaptic plasticity, and cognitive disabilities.

#### 4.2.2. Trisomic Genes and Neurogenesis Impairment

A number of trisomic genes in DS have been proposed to play a role in the proliferation impairment found in this condition. One of the genes overexpressed in the DS brain is DYRK1A (dual-specificity tyrosine-(Y)-phosphorylation regulated kinase 1A), an orthologous gene to the Drosophila gene minibrain [121]. DYRK1A codes for a serine-threonine protein kinase [122], which has important transcription factors as substrates and, consequently, appears to be implicated in multiple biological pathways. DYRK1A is essential for normal postembryonic neurogenesis [123, 124]. This gene plays a role in neuronal progenitor proliferation, neurogenesis, and neurodifferentiation, and regulates neuronal development, brain volume and cellular density in different brain areas [124–127]. The DYRK1A protein also modulates CREB (cAMP response element-binding protein) activity, which participates in synaptic plasticity signal transduction pathways [126]. Overexpression of DYRK1A inhibits proliferation, induces premature differentiation of neural progenitor cells in the developing mouse cerebral cortex, and impairs G1/G0-S phase transition in rat hippocampal progenitor cells [128, 129]. Recent studies have suggested that the DYRK1A gene could be a potential therapeutic target in DS because the inhibition of DYRK1A expression rescued several DS relevant phenotypes. Ortiz-Abalia et al. [130] demonstrated that the normalization of the Dyrk1A expression in the striatum of TgDyrk1A mice, through the injection of an adeno-associated virus type 2-mediated Dyrk1A RNA inhibitor (AAVshDyrk1A), rescued motor alterations in these animals.

The Olig1 and Olig2 genes are also overexpressed in DS individuals. These genes encode transcription factors that have been implicated in neurogenesis and oligodendrogenesis [131–133]. Chakrabarti et al. [103] have demonstrated the implication of these genes in the regulation of the number of inhibitory neurons during embryonic development. The normalization of Olig1 and Olig2 expression in Ts65Dn mice rescued the abnormal interneuron production and the balance between excitatory and inhibitory transmission [103].

The APP gene is triplicated in DS and in most DS mouse models, and it is thought to play a role in different DS phenotypes, such as the development of AD pathology. It has been proposed that the APP gene may also be involved in the altered neurogenesis characteristic of the trisomic condition. Trazzi et al. [115] have recently related increased levels of the APP fragment AICD to the overexpression of the negative regulator of the Shh pathway, Ptch1, in Ts65Dn mice neural precursors and to their proliferation impairment. APP overexpression may also alter the differentiation of newly born cells by acting upon the Notch pathway, which is implicated in the acquisition of a glial phenotype [134]. Notch is upregulated in the cortex of DS and AD patients and in DS fibroblasts [135]; therefore, it could shift the balance towards a glial phenotype rather than a neuronal phenotype in newly born cells.

#### 4.2.3. Therapies Targeting Neurogenesis

The implication of adult neurogenesis defects in DS-related cognitive impairments suggests that therapies targeted to rescue neurogenesis may be of value in treating intellectual disability in DS individuals.

The selective serotonin reuptake inhibitor fluoxetine is an antidepressant that has been shown to increase neurogenesis in the mouse DG and SVZ [113, 136]. Chronic treatment with fluoxetine restored neurogenesis in adult Ts65Dn mice [116]. Bianchi et al. [54] have recently shown that Ts65Dn mice treated with fluoxetine during the first two weeks of postnatal life showed rescued proliferation in the DG and SVZ, differentiation, and survival. Furthermore, this treatment restored brain derived neurotrophic factor (BDNF) expression, total granule cell number, and cognitive performance in a contextual fear conditioning task.

Another drug that markedly increases neurogenesis in the DG of adult normal mice is lithium, a drug prescribed for the treatment of bipolar depression [113]. Lithium treatment for 1 month rescued neurogenesis in the SVZ of 12-month-old Ts65Dn mice [114].

As mentioned above, the Shh pathway plays a key role in granule precursor cell (GPC) proliferation. Drugs targeting this pathway rescue neurogenesis alterations. Treatment of Ts65Dn mice with an activator of the Shh pathway, SAG 1.1, increased mitosis, restored cerebellar granule cell precursor populations [93], and rescued the cell proliferation of neural progenitors from the SVZ and DG [115]. Furthermore, a single injection of SAG 1.1 to newborn Ts65Dn mice restored cognition in these mice when they became adults [137].

Active care programs are one of the most successful therapeutic interventions used in DS individuals. In rodents, environmental enrichment has been associated with morphological, physiological, and cognitive improvements. These changes include increases in cortical weight and thickness, hippocampal neurogenesis, dendritic branching, length, number of dendritic spines and size of dendritic spines [138–140], facilitation of long-term potentiation [141, 142], and more efficient learning during different tasks [143–145].

Exposure of Ts65Dn mice to environmental enrichment for 7 weeks modulated spatial memory in a sex-dependent manner [55]. Environmental enrichment improved the performance of Ts65Dn females in the Morris water maze but lowered the performance of Ts65Dn male mice. In a subsequent study [42], it was shown that the deterioration found after environmental enrichment in Ts65Dn males was likely due to the stress induced by increased intermale aggression when the animals were housed in large groups. Enriching TS males in large groups (8–10) produced a large deterioration of performance in the Morris water maze and an increase in corticosterone plasma levels, effects that were not found when TS mice were housed in standard laboratory conditions or enriched in groups of 2-3.

Chakrabarti et al. [146] have recently shown that environmentally enriching groups of 2-3 Ts65Dn mice per cage increased cell proliferation and neurogenesis in the DG and SVZ of both male and female mice. It was proposed that this cellular response could underlie the cognitive improvements seen after special care programs in DS individuals.

Voluntary exercise is beneficial for cognition in both normal rodents and mouse models of altered cognition [110, 140, 147–149]. It has been suggested that these beneficial effects could be mediated, at least in part, by enhanced hippocampal neurogenesis [148, 150]. We have demonstrated that voluntary physical exercise improved the performance of TS mice in the Morris water maze but did not restore the neuromorphological phenotype (neurogenesis and hypocellularity in the DG), which suggests that the cognitive improvements produced by exercise were not mediated by neurogenesis-dependent mechanisms [83].

### 4.3. Apoptosis

Apoptosis or programmed cell death is physiologically involved in nervous system development and aging. It has been proposed that the hypocellularity found in DS brains could also be due to increased cell death. However, thus far, studies on the apoptotic processes in the trisomic condition have led to contradictory results. Some groups have reported increases in the number of apoptotic cells in DS brains [60, 151] and in Ts65Dn [89] and Ts1Cje [105] mice. In addition, changes in apoptotic regulatory proteins in different structures of DS brains have been found [152–156]. However, other studies have failed to find differences or have shown a reduced rate of apoptotic cell death in human and mouse trisomies [89, 157]. We have recently shown a downregulation of the antiapoptotic Bcl-Xl protein in the hippocampus of adult Ts65Dn mice, without changes in other pro- or antiapoptotic proteins in the cortex or the hippocampus [158]. Furthermore, we did not find any evidence of changes in molecular or cellular markers of apoptosis, suggesting that programmed cell death is not likely to play a role in the hypocellularity found in these mice brains.

### 4.4. Dendritic Hypotrophy

Altered synaptic plasticity is an additional mechanism that may underlie intellectual disability in DS individuals. Synaptic plasticity includes alterations in the number and the functional characteristics of synapses, which are mostly localized to dendrites and dendritic spines [159, 160].

Numerous studies have demonstrated impaired dendritic morphology in trisomic conditions. Although normal or even increased dendritic branching has been reported in DS fetuses and neonates [161–163], in DS children, neurons of the motor [164], visual [163, 165], and parietal cortex [166] show dendritic hypotrophy. These anomalies continue throughout the lifespan; in DS adults, the visual cortex, CA1, and CA3 are characterized by the presence of dendrites of shorter length and dendritic trees with reduced branching that progressively degenerate [161, 165, 167, 168]. Dendritic spines also show altered morphologies in the DS brain. Starting from infancy, spines are less numerous and smaller [168, 169], and their density is reduced to a greater extent in older DS individuals with AD [167, 170].

Mouse models also resemble the dendritic pathology of DS. In adult Ts65Dn mice, neocortical pyramidal neurons show a reduction in the length and arborization of dendrites and in the density of spines [171]. Spine density is also reduced in the granule cells of the DG in Ts65Dn, Ts1Cje, Ts2Cje, and Ts1Rhr mice [21, 48, 52, 172, 173]. In addition, in Ts65Dn, Ts1Cje, and Ts2Cje mice, these DG spines are characterized by several morphological anomalies, including an increase in the size of the heads and a decrease in the length of the necks [21, 52, 172]. Ts1Rhr mice also display enlarged spine heads, but no changes have been found in the morphology of spine necks of neurons in the cortex or the DG [174].

#### 4.4.1. Therapies Targeting Dendrites and Dendritic Spines

These anomalies in dendritic tree arborization, spine density, and morphology lead to reduced density of synapses and compromised synaptic function in DS individuals. Several groups have tested the value of various therapeutic strategies for rescuing dendritic pathologies.

Based on the observation that fluoxetine favors dendritic development in normal animals [175], Bianchi et al. [54] reported that the early administration of fluoxetine (P3–P15) restored dendritic maturation and dendritic branching of DG neurons in Ts65Dn mice. As mentioned above, this drug also rescued neurogenesis and cognitive deficits in this mouse model.

The enhancement of dendritic arborization and spine density has been firmly established as one of the positive effects of environmental enrichment [138, 139, 176]. Because environmental enrichment has been shown to improve cognition in female but not male Ts65Dn mice [55], Dierssen et al. [171] tested the effect of this experimental protocol on dendritic morphology. The authors found that the enriched environment increased dendritic length and spine density in the basal dendritic trees of the neocortical pyramidal cells of euploid animals but had no effect on Ts65Dn mice [171]. Thus, enhancements in dendritic arborization and spine density do not appear to be the mechanism by which enrichment improves cognition in Ts65Dn females.

### 4.5. Synaptic Pathology

As predicted by the reduced length and number of branches of dendrites and density of spines, the trisomic condition is characterized by a reduced number of synaptic contacts and alterations in synaptic plasticity. Ts65Dn mice show a reduction in synaptic density in the neocortex and CA1 at P9 [67] and in the hippocampal DG, CA1, and CA3 regions in adulthood [88]. However, the size of presynaptic boutons and the average length of synaptic clefts are increased in the cortex and hippocampus of Ts65Dn and Ts1Cje mice [52, 69, 172].

Not only the number and characteristics of synapses but also the relative distribution of different types of synapses is altered in trisomic mice, leaving the balance between excitatory and inhibitory synapses shifted toward increased inhibition in the trisomic brain. Ts65Dn mice have less excitatory (asymmetric) synapses in the temporal cortex, DG, CA1, and CA3 [88, 177], and glutamatergic synapses are reduced in the hippocampus of Ts65Dn mice [90]. An increased number of inhibitory synapse markers [172] have been reported in the DG of these mice, but no changes in the number of symmetric or asymmetric synapses were found in the fascia dentata of Ts65Dn mice [69]. Ts65Dn and Ts1Cje brains also show a redistribution of inhibitory synapses, with a relative decrease in inputs to the dendritic shafts and an increase in inputs on spine necks [52, 172]. An increased number of GABAergic interneurons in the somatosensory cortex of Ts65Dn mice have been reported [178], which implies an enhancement of inhibitory synapses. Finally, Chakrabarti et al. [103] have found enhanced neurogenesis of inhibitory neurons in the forebrain of Ts65Dn mice, which led to an increased inhibitory drive.

Overall, these morphological and functional disturbances compromise the physiological properties of synapses, possibly leading to cognitive impairments in DS and trisomic mice.

## 5. Electrophysiological Alterations in DS and Mouse Models of DS

DS individuals present small electroencephalographic (EEG) abnormalities. EEG alpha activity is relatively preserved in young individuals with DS, but older patients with dementia present abnormal activity [179]. EEG coherence differences [180] and alterations in event-related brain potentials (ERPs) have also been reported in DS individuals [181].

In mouse models of DS, altered synaptic plasticity in the hippocampus has been consistently reported. Hippocampal long-term potentiation (LTP) is considered to be the electrophysiological substrate of learning. Ts65Dn mice display reduced LTP in the hippocampal CA1 and DG regions [52, 182–186]. Similarly, Ts1Cje, Ts1Rhr, Dp(16)1Yey/+, and Tc1 mice show reduced hippocampal LTP [28, 30, 31, 48, 52]. However, Dep(17)1Yey/+ mice showed enhanced LTP [28].

It has been proposed that altered synaptic plasticity in the hippocampus of DS mouse models results from increased inhibition due to unbalanced excitatory and inhibitory neurotransmission [172, 186, 187]. Reduced activation of NMDA receptors is thought to hinder LTP induction in trisomic mice [30, 48, 52, 186]. Enhanced hippocampal long-term depression (LTD) has also been reported in the Ts65Dn mouse [183]. Scott-McKean and Costa [188] have demonstrated that increased cerebellar LTD, mediated by exaggerated NMDAR-dependent mechanisms, could be rescued by the administration of the NMDA receptor antagonist memantine.

In addition, overinhibition in the hippocampus of Ts65Dn mice has also been shown to be dependent on GABA_A_ receptors [172] because the GABA_A_ antagonist picrotoxin rescued the reduction in LTP induced by theta-burst stimulation (TBS) in these mice [184]. Furthermore, Kleschevnikov et al. [189] have shown that both GABA_A_ and GABA_B_ receptor-mediated components of evoked inhibitory postsynaptic currents (IPSCs) were significantly higher in Ts65Dn mice, suggesting an increase in presynaptic release of GABA. Thus, both GABA_A_ and GABA_B_ receptors are implicated in the reduced synaptic efficiency found in the DG of Ts65Dn mice.

The G-protein-activated inwardly rectifying potassium channel 2 (Girk2) gene is overexpressed in DS individuals. Girk channels generate a GABA_B_ receptor-dependent slow inhibitory postsynaptic potential in hippocampal neurons [190]. It has been proposed that an increase in Girk2 gene expression may produce overinhibition in hippocampal neurons and contribute to LTP failure in the trisomic condition [191].

### 5.1. Therapies Targeting Overinhibition

Because overinhibition in the trisomic brain appears to underlie LTP impairments and, therefore, alter learning and memory processes, a number of studies have tested drugs that reduce GABA-mediated inhibition in an attempt to rescue the electrophysiological substrates of cognition.

It is well established that the GABA_A_ receptor system plays an important role in cognition. Nonselective positive modulators of the GABA_A_ receptor disrupt learning and memory processes [192–194], while nonselective negative modulators improve cognitive processes [195–197]. Reducing inhibition in the Ts65Dn brain by administering the GABA_A_ antagonists picrotoxin (PTX), bilobalide (BB), or pentylenetetrazole (PTZ) restored LTP and cognition in the object recognition test in these mice [185]. Rueda et al. [198] confirmed that chronic PTZ treatment also rescued Ts65Dn mice performance in the Morris water maze.

However, non-selective GABA_A_ negative modulators cannot be safely used to improve cognition due to their anxiogenic and proconvulsant effects [199]. Among the different GABA_A_ receptor subtypes, GABA_A_ *α*5 subunit-containing receptors are known to facilitate cognition in hippocampal-dependent tasks [200, 201]. Moreover, selective GABA_A_ *α*5 negative allosteric modulators, also called inverse agonists, have cognition-enhancing effects without anxiogenic or proconvulsant side effects [202–204]. A functionally selective GABA_A_ *α*5 inverse agonist, *α*5IA, has been shown to rescue learning and memory deficits in TS mice without inducing anxiogenic and convulsant side effects [205].

Further support for the efficacy of reducing GABA-mediated overinhibition to improve cognition in trisomic mice comes from a recent report that demonstrated that environmental enrichment reduced the release of GABA in the hippocampus and visual cortex of Ts65Dn mice while rescuing spatial learning and hippocampal LTP [206].

## 6. Altered Neurotransmission and Receptors

Alterations in several neurotransmitters and changes in the expression and function of their receptors, in both DS individuals and mouse models of this condition have been demonstrated. These impairments may be responsible for other phenotypes found in trisomic conditions, such as defects in neurogenesis, synaptic transmission, and cognition. Dopamine, taurine, and histamine levels have been shown to be altered in the brains of DS fetuses and adults [97, 98, 207–209]. The main neurotransmitter and receptor alterations in DS and in the Ts65Dn mouse model are summarized in Table 4.

### 6.1. GABA

GABA is reduced in DS fetuses [207]. However, as predicted from the enhanced inhibition of the trisomic brain, an increase in the number of inhibitory neurons has been found in Ts65Dn mice due to the overexpression of the Olig1 and Olig2 genes (see above) [103, 178]. Furthermore, it has been suggested that enhanced presynaptic GABA release may be responsible for the increased hippocampal inhibitory postsynaptic potentials (IPSPs) observed in these mice [189].

A number of alterations have been reported in the expression of various GABA receptor subunits. In neurospheres from fetuses with DS, upregulation of the *α*2 and downregulation of the *α*5 and *β*3 subunits of the GABA_A_ receptor have been reported [210]. In the hippocampus of Ts65Dn mice, reductions in the number of *β*2 and *β*3 subunits of the GABA_A_ receptor were found [69]. Brain synaptosomes of Ts65Dn mice show a reduction in GABA_A_ *α*1 receptor expression [211]. Changes in the R1 subunit of the GABA_B_ receptor have also been reported in Ts65Dn mice [69]. However, Kleschevnikov et al. [189] did not find changes in the levels of GABA_A_ or GABA_B_ receptor subunits by western blot analysis.

GABA_A_ activity is known to regulate neuronal proliferation, migration, differentiation, and integration of newly generated neurons [212–214]. The enhanced GABA_A_-mediated inhibition shown by Ts65Dn mice could, therefore, be implicated in the alterations in neuronal proliferation and survival found in these mice.

### 6.2. Excitatory Transmitters

Increased inhibition in the trisomic condition is also caused by alterations in excitatory transmission. Although similar levels of glutamate [215] are found in fetuses with and without DS, decreased levels of aspartate and glutamate have been found in several areas of the adult DS brain [97, 98, 208].

As detailed above, altered hippocampal LTP in trisomic mice suggests disturbances in NMDA receptor signaling. In Ts65Dn mice, a reduction of the GluR1 subunit of the AMPA receptor [69] and of the NR2A and NR2B subunits of the NMDA receptor [216] has been reported. However, other studies failed to find changes in the GluR1 subunit in brain homogenates or changes in the NR2A and NR2B subunits in synaptosomes of these mice [211].

Ts65Dn and Ts1Cje mice exhibit hypersensitivity to the locomotor stimulatory effect of MK-801, an NMDA receptor channel blocker [217].

Alterations in the signaling mechanisms downstream of the NMDA receptor have also been reported; the hippocampi of Ts65Dn mice show disturbances in the calcium/calmodulin-dependent protein kinase II (CaMKII), phosphatidylinositol 3-kinase (PIP3K)/Akt, extracellular signal-regulated kinase (ERK), protein kinase A (PKA), and protein kinase C (PKC), all of which have been shown to be involved in synaptic plasticity [218].

One of the targets of the NMDA receptor is the protein phosphatase calcineurin (CaN). The DSCR1 gene encodes a protein that inhibits CaN, and this gene is overexpressed in the Ts65Dn brain [219]. The inhibition of CaN activity increases the mean open time and opening probability of the NMDA receptor [220]. Memantine, a partial agonist of the NMDA receptor, often prescribed for the treatment of AD-dementia, acts as an open-channel blocker and has been proposed to mimic the actions of CaN and restore the function of the NMDA receptor. Costa et al. [221] demonstrated that the acute administration of memantine improved contextual fear conditioning in Ts65Dn mice. Chronic treatment with memantine also improved Ts65Dn mice performance in the Morris water maze [90] and in the novel object recognition test and water radial arm maze [222]. Memantine slightly reduced brain APP levels and normalized the levels of hippocampal excitatory synapses in Ts65Dn mice [90]. However, memantine did not rescue Ts65Dn morphological alterations, as the number of hippocampal granule [90], basal forebrain cholinergic, and locus coeruleus neurons [222] remained low in memantine-treated Ts65Dn mice. Nevertheless, these mice showed increased BDNF levels in the hippocampus and the prefrontal cortex.

In spite of the rescue induced by memantine of several DS-relevant phenotypes in the Ts65Dn mouse, a recent randomized double-blind clinical trial failed to find any benefit of memantine administration for 52 weeks on cognitive impairment and dementia in DS individuals over 40 years of age [223].

### 6.3. Serotonin

Deficits in serotonin (5-HT) have been reported in the frontal cortex of DS fetuses [207] and in adult DS brains [97, 98, 209]. However, Ts65Dn mice show unchanged levels of 5-HT in the hippocampus [54], and no alterations were found in the histological analysis of serotonergic neurons of the dorsal and medial raphe nuclei of these mice [224]. 5-HT has a role in neurogenesis, neuronal differentiation, dendritic development, axon myelination, and synaptogenesis [225]. Thus, the reduction of this transmitter in DS fetal and adult brains may underlie a number of altered neuromorphological and cognitive phenotypes.

The 5-HT_1A_ receptor has also been implicated in the regulation of neurogenesis [113, 226, 227]. Reduced levels of the 5-HT_1A_ receptor have been reported in the DS brain at birth [228], in hippocampal neurospheres, and in the hippocampus of newborn Ts65Dn mice [54]. Thus, reduced 5-HT_1A_ receptor expression may underlie the defective neurogenesis found in Ts65Dn mice [54]. Moreover, treatment with the 5-HT_1A_ reuptake inhibitor fluoxetine rescued the expression levels of this receptor in Ts65Dn mice, suggesting that this effect may underlie the rescue of proliferation produced by this drug, as previously mentioned.

### 6.4. Acetylcholine

One of the anatomical substrates of learning and attention is the septohippocampal cholinergic system [95, 96]. A number of studies have demonstrated alterations of this system in the trisomic condition. Deficits in the cholinergic system have been found in DS fetuses [207], and choline acetyltransferase (ChAT) activity is reduced in the brains of adults with DS [97, 98].

Although a normal number of cholinergic neurons is found in young Ts65Dn mice, basal forebrain cholinergic neurons (BFCNs) degenerate with age in these mice [99–102]. However, ChAT activity is increased in the cortex and hippocampus of 10-month-old Ts65Dn mice, likely in an attempt to compensate for the reduced number of cholinergic neurons [82, 100, 229, 230].

### 6.5. Noradrenaline

The levels of noradrenaline (NA) are normal in DS fetuses [207] but are reduced in adult DS brains [97, 98], likely as a consequence of the neurodegeneration of the locus coeruleus [231, 232]. Ts65Dn mice also show a loss of locus coeruleus neurons starting at 6 months of age [53].

Ts65Dn mice show unchanged numbers of *β*-adrenoceptors in the cortex and hippocampus; however, their function is altered. Basal production of cyclic AMP in the hippocampus of TS mice was impaired. In addition, the responses of adenylyl cyclase to the stimulation of *β*-adrenoceptors with isoprenaline and of the catalytic subunit with forskolin were both severely depressed [233, 234]. Aging DS brains also show a dramatic reduction in basal and stimulated cAMP production [235].

NA has been shown to play a role in neurogenesis, as neuron proliferation is enhanced or impaired following increases or reductions in NA transmission, respectively [236]. Therefore, altered NA transmission in the trisomic condition may also play a role in the impairment of adult hippocampal neurogenesis. Furthermore, a link between noradrenergic afferents from the locus coeruleus to hippocampal neurons and contextual learning has been demonstrated [237]. This hippocampal-dependent cognitive process is impaired in individuals with DS [6] and in Ts65Dn mice [53]. A recent study by Salehi et al. [53] demonstrated that enhancing NA transmission through the administration of L-Threo-3, 4-dihydroxyphenylserine (L-DOPS), a synthetic amino acid that is metabolized by NA-containing neurons to produce NA, or xamoterol, a ß1-adrenergic receptor partial agonist, rescued contextual learning in Ts65Dn mice. These authors hypothesized that, given the finding that NA can activate or inhibit GABAergic neurons and that GABA can increase the release of NA, there could be an overlap in the mechanisms by which GABA_A_ antagonists and NA-enhancing drugs improve learning in Ts65Dn mice.

### 6.6. Neurotrophins

The role of neurotrophins (NT) in neuronal survival, differentiation, migration, and synaptic plasticity is well documented [238–240]. Consequently, alterations in their expression may alter many aspects of neurodevelopment.

The reduced expression of BDNF has been observed in the hippocampus of DS fetuses [241], and the reduced expression of both BDNF and the tyrosine kinase receptor TrkB has been observed in the cerebral cortex of DS fetuses [242]. Young Ts65Dn mice also show reduced BDNF levels in the hippocampus [54, 243] and in the frontal cortex during adult stages [244]. Because BDNF has a role in neuronal survival and differentiation [213, 215], it is a natural target for several treatments to restore neurogenesis in the trisomic brain. In Ts65Dn mice, fluoxetine restored BDNF expression, survival of newborn cells, differentiation, and granule cell number.

NT-3 is increased in the hippocampus of newborn and adult Ts65Dn mice [245], potentially in an attempt to compensate for the neuronal loss found in these mice.

Nerve growth factor (NGF) is generated in the hippocampus and retrogradely transported to the soma of BFCNs [239]. NGF levels are reduced in the hippocampus of young Ts65Dn mice [50], and the retrograde transport of NGF to the basal forebrain is hindered in older Ts65Dn and Ts1Cje mice [50, 100]. NGF enhances the survival, differentiation, and maintenance of neurons, including BFCNs [239]. The administration of NGF to Ts65Dn mice rescued the altered size and number of BFCNs [100].

Peptide 6, an active region of ciliary neurotrophic factor (CNTF), modulates the CNTF pathway by inhibiting the antineurogenic activity of leukemia inhibitory factor, thereby increasing neurogenesis [246]. Administration of peptide 6 to Ts65Dn mice reduced learning and memory deficits, enhanced the pool of neural progenitor cells in the hippocampus, and increased the level of synaptic proteins crucial for synaptic plasticity [247].

Considering the role of Dyrk1A in neuronal progenitor proliferation, neurogenesis, and neurodifferentiation, it has been suggested that molecules targeting this gene could provide therapeutic benefits to DS phenotypes. Epigallocatechin gallate (EGCG), an antioxidant extracted from green tea, is an inhibitor of the protein kinase DYRK1A [248].

The chronic administration of EGCG from conception to adulthood rescued BDNF levels in the hippocampus of Dyrk1a transgenic mice [241]. Concomitant to this neurotrophic factor normalization, these mice presented an increase in brain volume and improved cognitive performance. Other studies have demonstrated that the acute administration of EGCG normalizes hippocampal LTP in Ts65Dn mice [249]. However, EGCG affects a wide array of signal transduction pathways including the MAPK, PI3K/AKT, Wnt, and Notch pathways [250], which are altered in Ts65Dn mice [135]; thus, its beneficial effects could be mediated by mechanisms different from Dyrk1A inhibition.

Finally, Fukuda et al. [243] have recently demonstrated that the chronic administration of the analgesic neurotrophin to Ts65Dn mice prevents the age-dependent decline in hippocampal BDNF expression. This treatment also enhanced the performance of these mice in the radial arm maze. It has been proposed that the analgesic action of neurotrophin is mediated by the noradrenergic and GABAergic systems [251]; therefore, the cognitive-enhancing effects could also be determined by improvements in the function of these transmitter systems.

## 7. Neurodegeneration

Although neurodevelopmental alterations occurring from early embryonic stages are likely to cause intellectual disability, there are a number of neurodegenerative mechanisms in DS that complicate this scenario. Atrophy of a number of structures, including the hippocampus, amygdala [71, 252], the corpus callosum, and the parietal, frontal, and occipital cortices [77, 78], has been reported in nondemented adult DS brains. Furthermore, neuroinflammation, increased oxidative stress, and the development of AD neuropathology are hallmarks of DS (Table 5).

### 7.1. Neuroinflammation

DS and AD brains are characterized by activated microglia, and increased levels of proinflammatory cytokines that lead to neuroinflammation are likely involved in neurodegeneration [253, 254]. The activation of microglia may play a role in the loss of basal forebrain cholinergic neurons in Ts65Dn mice.

Minocycline is a semisynthetic tetracycline that inhibits neuronal death and reduces inflammatory activity by blocking inflammatory mediators [255]. The chronic administration of minocycline to adult Ts65Dn mice inhibits microglia activation in the basal forebrain and hippocampus, prevents the loss of cholinergic neurons in the medial septal nucleus, attenuates the loss of hippocampal calbindin-positive neurons, and improves working and reference memory in these mice [102].

### 7.2. Neuropeptides

Vasoactive intestinal peptide (VIP) is neuroprotective, as it promotes the release of several survival factors from astrocytes and regulates neuropeptide release from glial cells, including activity-dependent neuroprotective protein (ADNP) and activity-dependent neurotrophic factor (ADNF) [256]. The active peptide fragments of ADNP and ADNF, NAPVSIPQ (NAP), and SALLRSIPA (SAL) have been shown to protect neurons from oxidative stress and limit the severity of traumatic head injuries, stroke, and the toxicity associated with the A*β* peptide [257, 258].

In cultures of DS cortical neurons, treatment with SAL or NAP increases neuronal survival [259]. In Ts65Dn mice, prenatal treatment with these two peptides rescued the acquisition of neurodevelopmental milestones [260], increased the reduced levels of ADNP, and normalized the levels of the NMDA receptor subunits NR2A, NR2B, and the GABA_A_ receptor subunit *β*3 [216]. Furthermore, subchronic treatments with D-NAP and D-SAL to adult Ts65Dn mice rescued learning and memory and ADNP and NRD2 levels [256].

### 7.3. Oxidative Stress

In DS individuals and in the Ts65Dn mouse, there is an overexpression of SOD1, the gene responsible for the formation of superoxide dismutase, an enzyme that modifies oxygen free radicals into hydrogen peroxide. The overproduction of hydrogen peroxide leads to the overproduction of highly reactive oxygen free radicals, which damage cell membranes, including the mitochondrial membrane, and deteriorate lipids, proteins, and mitochondrial DNA. This set of alterations is called oxidative stress. Evidence for increased mitochondrial superoxide production in DS individuals has been repeatedly demonstrated [261, 262]. Therefore, in this condition, some cells are under the permanent threat of oxidative stress with mitochondrial damage, which deteriorates cell life, facilitating aging, and death. This increase in oxidative stress occurs during pre- and postnatal development. Increased oxidative stress in the fetal stages can modify processes such as neurogenesis, differentiation, migration and net connexion, as well as survival [261, 263–265].

In an attempt to reduce oxidative stress-induced neurodegeneration, several groups have tested the efficacy of various antioxidants to reduce the altered phenotypes in Ts65Dn mice. SGS-111, an analogue of the nootropic piracetam, has been shown to increase neuronal survival and prevent the accumulation of intracellular free radicals, peroxidative damage, and the development of neurodegenerative changes in both normal and DS cultured neurons [265]. However, the chronic administration of SGS-111 to Ts65Dn mice from conception to adulthood did not rescue their cognitive alterations [266]. Conversely, the administration of another antioxidant, vitamin E, to Ts65Dn mice during adult stages [267] or from conception throughout their entire life [268] reduced markers of oxidative stress, improved cognitive performance, reduced cholinergic neuron pathology in the basal forebrain, and increased cell density in the DG.

A recent report [269] revealed a positive effect of folinic acid on developmental age in children with DS. Folinic acid has an antioxidant effect and is known to be involved in CNS development. Folate deficiency causes neurological, psychiatric, and cognitive disorders, and DS probably involves either folate deficiency or defective folate use [270]. However, in a randomized controlled trial, Ellis et al. [271] failed to find any efficacy of antioxidants and folinic acid supplementation in children with DS cognitive development. In addition, a number of studies on the effects of antioxidant supplementation in children and adults with DS did not find any benefit of this treatment on cognition. Salman [272] performed a systematic review of eleven randomized controlled trials on the effects of dietary supplements (vitamins and/or minerals) on cognitive function in subjects with DS. None of these trials reported cognitive enhancing effects in individuals with DS. Moreover, in a two-year randomized, double blind, placebo-controlled trial daily oral antioxidant supplementation (*α*-tocopherol, ascorbic acid, and the mitochondrial cofactor: *α*-lipoic acid) did not produce any improvement in cognitive functioning nor a stabilization of cognitive decline in adults with DS [273].

### 7.4. Estrogens

Because estrogens maintain the function of basal forebrain cholinergic neurons, it has been proposed that the administration of estrogens may be useful in reducing the loss of these neurons in AD and DS individuals [274].

The chronic administration of estrogens to aged female Ts65Dn mice enhanced cognition, increased the size and number of cholinergic neurons, increased the levels of NGF in the medial septum [275], restored the number of cholinergic terminals in hippocampus, and restored the levels of the dendritic marker Map2 [276].

### 7.5. AD Neuropathology

One of the genes triplicated in the trisomic condition is APP. In DS individuals, the increased expression of this gene leads to the increased production of *β*-amyloid, which is thought to be responsible for the amyloid plaque pathology and degeneration of BFCNs found in 100% of DS individuals over 40 years of age. Ts65Dn mice also show age-related elevations in the levels of the APP protein [277] and the *β*-amyloid peptide [278] in the cortex and hippocampus. In these mice, the overexpression of APP has also been implicated in the degeneration of both the cholinergic and noradrenergic neurons that provide strong modulatory inputs to the hippocampus [279]. Thus, this age-related noradrenergic and cholinergic deafferentation is likely compromising hippocampal function.

To test the effect of *β*-amyloid reductions on the Ts65Dn mice altered phenotypes, Netzer et al. [278] administered the gamma secretase inhibitor DAPT (N-[N-(3,5-Difluorophenacetyl)-L-alanyl]-S-phenylglycine t-butyl ester). This treatment reduced *β*-amyloid levels and rescued spatial learning in these mice. Because *β*-amyloid is a regulator of the glutamatergic system, the authors proposed that the cognitive enhancing effects of DAPT could be mediated by an enhancement and/or a regulation of excitatory synaptic transmission.

Given the role of the cholinergic system in cognition and the degeneration of this system in AD and DS individuals, it has been proposed that pharmacological enhancements of this system could help diminish cognitive deterioration in these conditions. Donepezil is an acetylcholinesterase inhibitor that is widely prescribed to enhance cholinergic transmission in the treatment of AD dementia. However, the chronic administration of donepezil did not improve learning and memory in Ts65Dn mice [198]. Similarly, donepezil administration to young adult individuals with DS has produced ambiguous results [280–283].

Piracetam is a drug that shows cognitive-enhancing effects in patients with a number of cognitive disorders and dementia [284] and in several animal models. Although the mechanisms underlying these effects are not known, it has been proposed that piracetam may be enhancing cholinergic and modulating glutamatergic transmission [284]. However, piracetam treatment did not improve cognitive impairments in children with DS [285] or in the Ts65Dn mouse [286].

## 8. Concluding Remarks

The first partial trisomic models, the Ts65Dn and Ts1Cje models, demonstrated that DS phenotypes could be recapitulated in mice. More recently, knockout and transgenic mice for individual genes and new animals that are trisomic for different regions of orthologues of Hsa21 regions are helping to identify dosage-sensitive genes involved in DS phenotypes. Although some of these triplicated genes may play a role individually, it appears that DS phenotypes arise from the complex effects of groups of Hsa21 genes.

In the last 20 years, the characterization of these animal models of DS, particularly the Ts65Dn mouse, has been enormously useful to understand of the neurobiological basis of intellectual disability. Several mechanisms have been proposed to underlie this altered cognition, including impaired neurogenesis leading to hypocellularity in the cortex, hippocampus, and cerebellum, altered dendritic morphology, altered synapses, increased inhibition and neurodegeneration. The new knowledge of the pathogenic mechanisms in DS individuals has been applied to the development of new pharmacotherapies. Several drugs have been shown to rescue neurogenesis, hypocellularity, electrophysiological deficits, and cognitive alterations in the Ts65Dn mouse. These studies provide the basis for developing clinical trials in DS individuals and sustain the hope that some of these drugs will be useful in rescuing intellectual disability in DS individuals.

## Figures and Tables

**Table 1:** Behavioural and cognitive alterations in DS and in the different DS mouse models.

Trisomy	Hsa21	Segment of Mmu16						Segment of Mmu17		Segment of Mmu10	Segment of Mmu16, Mmu17, and Mmu10	Hsa21
	DS	Dp(16)1Yey/+	Ts65Dn	Ts2Cje	Ts1Cje	Ms1Ts65	Ts1Rhr	Dep(17)1Yey/+	Ts1Yah	Dp(10)1Yey/+	Dp(10)1Yey/+; Dp(16)1Yey/+; Dep(17)1Yey/+ Yu et al., 2010	Tc1
Motor skills	Delayed acquisition		Delayed acquisition									

Motor coordination	Impaired		Impaired									Impaired

Activity and attention	Reduced attention		Hyperactivity and reduced attention		Normal activity	Normal activity	Normal activity					Increased spontaneous activity

Context discrimination		Impaired	Impaired					Unchanged		Unchanged	Impaired	

Spatial learning and memory	Impaired	Impaired	Impaired		Impaired	Impaired		Unchanged	Enhanced	Unchanged	Impaired	Impaired

Working and reference memory	Impaired		Impaired									Impaired

Novel object recognition			Impaired				Impaired		Impaired			

Operant conditioning			Impaired									

**Table 2:** Neuromorphological and electrophysiological alterations DS and DS mouse models.

Trisomy	Hsa21	Segment of Mmu16						Segment of Mmu17		Segment of Mmu10	Segment of Mmu16, Mmu17, and Mmu10	Hsa21
	DS	Dp(16)1Yey/+	Ts65Dn	Ts2Cje	Ts1Cje	Ms1Ts65	Ts1Rhr	Dep(17)1Yey/+	Ts1Yah	Dp(10)1Yey/+	Dp(10)1Yey/+; Dp(16)1Yey/+; Dep(17)1Yey/+ Yu et al., 2010	Tc1
Brain volume	Reduced		Reduced	Reduced during the embryonic period	Reduced during the embryonic period	Reduced	Reduced at 4 months of age					

Neuronal density	Reduced		Reduced	Reduced	Not affected							

Cerebellar volume	Reduced		Reduced		Reduced	Not affected	Reduced					Reduced

Cerebellar neuronal density			Reduced		Reduced	Reduced						Reduced

Neurogenesis	(i) Impaired neural precursor proliferation		(i) Impaired neural precursor proliferation	(i) Impaired neural precursor proliferation	(i) Impaired neural precursor proliferation
	(ii) Slowing of the cell cycle		(ii) Slowing of the cell cycle		(ii) Impaired neurodifferentiation
	(iii) Impaired neurodifferentiation		(iii) Impaired neurodifferentiation		(iii) Impaired cerebellar neurogenesis
	(iv) Impaired cerebellar neurogenesis		(iv) Impaired cerebellar neurogenesis		

Dendrites and dendritic spines	(i) Impaired morphology		(i) Impaired morphology	(i) Impaired morphology	(i) Impaired morphology		(i) Impaired morphology					
	(ii) Reduced density		(ii) Reduced density	(ii) Reduced density	(ii) Reduced density		(ii) Reduced density					

Synaptic density			(i) Reduced		(i) Size of presynaptic boutons and average length of synaptic clefts are increased							
			(ii) Size of presynaptic boutons and average length of synaptic clefts are increased									

Inhibition			(i) Increased number of inhibitory synapses		(i) Redistribution of inhibitory synapses							
			(ii) Decreased number of excitatory synapses									
			(iii) Redistribution of inhibitory synapses									
			(iv) Increased number of GABAergic interneurons									

Electrophysiology	(i) EEG abnormalities	(i) Impaired hippocampal LTP	(i) Impaired hippocampal LTP		(i) Impaired hippocampal LTP		(i) Impaired hippocampal LTP	(i) Enhanced hippocampal LTP		(i) Unchanged LTP		(i) Impaired hippocampal LTP
	(ii) EEG coherence differences											
	(iii) Alterations in event-related potentials											

**Table 3:** Therapeutical approaches tested in DS and in the Ts65Dn mouse.

Therapies	DS	Ts65Dn
*Targeting neurogenesis*		
(i) Fluoxetine (ii) Lithium (iii) SAG 1.1 (iv) Environmental enrichment	(i) Not tested (ii) Not tested (iii) Not tested (iv) Improves cognition	(i) Restores BDNF levels, neurogenesis, dendritic maturation and branching and cognition (ii) Restores neurogenesis (iii) Restores neurogenesis and cognition (iv) Restores neurogenesis, improves cognition, no effect on dendritic arborization in TS mice

*Targeting inhibition*		
(i) Picrotoxin (ii) Bilobalide (iii) PTZ (iv) *α*5IA	(i) Not tested (ii) Not tested (iii) Not tested (iv) Not tested	(i) Rescues LTP and cognition (ii) Rescues LTP and cognition (iii) Rescues LTP and cognition (iv) Rescues cognition

*Targeting NMDA receptor functioning*		
(i) Memantine	(i) No effect	(i) Improves cognition, reduces APP levels

*Targeting NA functioning*		
(i) L-DOPS (ii) Xamoterol	(i) Not tested (ii) Not tested	(i) Rescues cognition (ii) Rescues cognition

*Targeting neurotrophins*		
(i) NGF	(i) Not tested	(i) Rescues BFCNs altered size and number
(ii) Peptide 6 (CNFT)	(ii) Not tested	(ii) Improves learning and memory, enhanced neurogenesis
(iii) EGCG	(iii) Not tested	(iii) Rescued BDNF levels, brain size, and LTP in the Dyr1A Tg mouse
(iv) Neurotrophin	(iv) Not tested	(iv) Prevents decline in BDNF expression, improves cognition

*Targeting inflammatory activity*		
(i) Minocycline	(i) Not tested	(i) Inhibits microglia activation, prevents neuron loss, improves working, and reference memory

*Neuropeptides*		
(i) NAP and SAL	(i) Not tested	(i) Rescues acquisition of neurodevelopmental milestones, increases ADNP levels and rescued ADNP levels

*Targeting oxidative stress*: *antioxidants *		
(i) SGS111 (ii) Vitamin E (iii) Combined antioxidant supplementation (iv) Folinic acid/ folinic acid + antioxidants	(i) Not tested (ii) No effect (iii) No effect (iv) Beneficial effect on developmental age/no effect	(i) No effect on cognition (ii) Reduced oxidative stress, improved cognitive performance, reduced cholinergic neuron pathology, and increased cell density in the DG

Estrogens	(i) Not tested	(i) In females enhanced cognition, increased the size and number of cholinergic neurons and NGF levels

*Targeting AD neuropathology*		
(i) DAPT (ii) Donepezil	(i) Not tested (ii) No effect/small effect	(i) Reduced beta-amyloid levels, rescued cognition (ii) No effect

**Table 4:** Neurotransmitter and receptor alterations in DS and in the Ts65Dn mouse model of Down syndrome.

	DS	Ts65Dn
GABA	(i) Reduced in fetuses	(i) Increased number of GABAergic interneurons

Excitatory transmitters	(i) Decreased levels of glutamate and aspartate in adults	(i) Alterations in the composition of the AMPA and NMDA receptor (ii) Alterations in the signalling mechanisms downstream the NMDA receptor

5-HT	(i) Deficits of 5-HT in the frontal cortex (ii) Reduced levels of the 5-HT_1A _ receptor	(i) Unchanged levels of 5-HT (ii) Reduced levels of the 5-HT_1A_ receptor

Ach	(i) Deficits in the cholinergic system and in ChAT activity	(i) Reduced levels of markers for Ach (ii) Increased ChAT activity

NA	(i) Reduced levels in adult brains (ii) Altered *β*-adrenoceptor function in aged brains	(i) Loss of locus coeruleus neurons starting at 6 months of age (ii) Altered *β*-adrenoceptor function

*Neurotrophins*		
(i) BDNF (ii) NT3 (iii) NGF	(i) Reduced levels in fetuses	(i) Reduced levels (ii) Increased levels (iii) Reduced levels

**Table 5:** Neurodegenerative processes in DS and in the Ts65Dn mouse.

	DS	Ts65Dn
Neuroinflammation	(i) Activated microglia and increased levels of proinflammatory cytokines	(i) Activated microglia

Oxidative stress	(i) Increased	(i) Increased

AD neuropathology	(i) Cholinergic neuron loss, plaques, and tangles	(i) Cholinergic neuron degeneration, increased APP and *β*-amyloid levels
